# Differences in the prevalence of cardiovascular and metabolic diseases coinciding with clinical subtypes of obstructive sleep apnea

**DOI:** 10.1002/clc.23941

**Published:** 2022-11-20

**Authors:** Yang Gao, Yaxin Guo, Jiajia Dong, Yifan Liu, Wen Hu, Mi Lu, Yueran Shen, Yi Liu, Yongxiang Wei, Zhenlin Wang, Xiaojun Zhan

**Affiliations:** ^1^ Department of Otolaryngology‐Head and Neck Surgery, Xuanwu Hospital Capital Medical University Beijing China; ^2^ Department of Otolaryngology‐Head and Neck Surgery, Beijing Anzhen Hospital Capital Medical University Beijing China; ^3^ Beijing Institute of Heart Lung and Blood Vessel Diseases Beijing China; ^4^ Department of Otorhinolaryngology‐Head and Neck Surgery Capital Institute of Pediatrics Beijing China

**Keywords:** cardiovascular and metabolic diseases, clinical subtypes, latent cluster analysis, obstructive sleep apnea

## Abstract

**Background:**

It is unclear about the cardiovascular and metabolic diseases (CMD) among Chinese patients with different clinical subtypes of obstructive sleep apnea (OSA).

**Hypothesis:**

The prevalence of CMD varies among OSA patients of different clinical subtypes.

**Methods:**

A total of 1483 Chinese patients with OSA were assessed to evaluate the existence of clinical subtypes of OSA using latent class analysis. We compared the differences in demographic characteristics and prevalence of CMD using ANOVA and *χ* ^2^ tests. Associations between clinical subtypes and disease prevalence were assessed using adjusted logistic regression.

**Results:**

We identified prevalent CMD in Chinese patients with the four subtypes of OSA: excessively sleepy (ES), moderately sleepy with disturbed sleep (ModSwDS), moderately sleepy (ModS), and minimally symptomatic (MinS). The ES subtype had a higher body mass index, average Epworth Sleepiness Scale score, Apnea‐hypopnea index, and oxyhemoglobin saturation below 90% compared with the other subtypes (*p* < .05). The MinS subtype had the lowest mean ESS score (*p* < .05). We found a significant difference in the prevalence of CMD among the four subtypes, with the highest proportion of cases of CMD in the ES subtype. In adjusted models, significant associations with CMD were also found. ES, ModSwDS, ModS, and MinS subtypes are very high‐risk, high‐risk, medium‐risk, and low‐risk in prevalent CMD.

**Conclusions:**

This study identified four clinical subtypes of OSA in Chinese patients. Each clinical subtype corresponds with a different level of prevalence of CMD; this finding is helpful for the more precise treatment of patients with different clinical manifestations.

## 1 INTRODUCTION

Obstructive sleep apnea (OSA) is a sleep and respiratory disease with a high risk of body damage and incidence rate.^1^, ^2^ A recent systematic analysis estimated that 936 million adults aged 30–69 years have mild to severe OSA, and the number of affected individuals is the highest in China.^1^ More than half of OSA cases are complicated due to cardiovascular and metabolic diseases (CMD), such as hypertension, coronary heart disease, arrhythmia, and stroke.^3^, ^4^, ^5^, ^6^

Continuous positive airway pressure (CPAP) is commonly used for the clinical treatment of OSA.^4^, ^7^, ^8^ However, it remains unclear whether patients with OSA benefit after this treatment; some studies^9^, ^10^ have found no benefit to the cardiovascular system following CPAP treatment. For example, the final results of the SAVE^9^ and ISAACC^10^ studies show that CPAP does not improve the prognosis of patients with OSA complicated with coronary heart disease. A follow‐up study^11^, ^12^ indicated that the difference in subtypes in OSA patients resulted in different cardiovascular prognoses and diverse responses to CPAP treatment. The clinical subtypes of sleep disorders in patients with OSA are associated with cardiovascular risk.^12^, ^13^

Based on sleep‐related symptoms, patients with OSA were divided into subtypes.^12^, ^13^, ^14^ Investigators found that excessively sleepy patients with OSA were more likely to have hypertension,^15^ and those with daytime sleepiness had a higher prevalence of hypertension and type 2 diabetes.^16^ In addition, excessive daytime sleepiness in patients with moderate and severe OSA is associated with a higher risk of major adverse cardiac events after myocardial infarction.^17^

Recently, many studies^12^, ^13^, ^18^ have shown that there are clinical subtypes of patients with OSA. With the goal of better understanding the heterogeneity of OSA clinical presentations in Chinese patients, this study applied latent cluster analysis (LCA) to identify subtypes of patients with OSA who experience distinct combinations of symptoms. We also observed and compared the differences in the prevalence of CMD according to clinical subtypes in patients with OSA.

## 2 METHODS

### 2.1 Participants

The current study had a cross‐sectional design and enrolled 1483 patients with OSA at the Sleep Center of Beijing Anzhen Hospital (see Supporting Information: Figure A.1). The inclusion criteria were as follows: over 18‐year‐old; diagnosed with OSA by portable monitor. The exclusion criteria were as follows: severely impaired consciousness; previous history of malignancy. Participants with central sleep apnea syndrome were also excluded from the study. The study protocol was approved by the institutional review board of Beijing Anzhen Hospital. Informed consent was obtained from all participants.

### 2.2 Clinical information

All participants were clinically evaluated, and the information recorded included sociodemographic characteristics (gender, age), and comorbidities (type 2 diabetes, hyperlipidemia, hypertension, coronary heart disease, arrhythmia, heart failure, stroke, asthma, chronic obstructive pulmonary disease, reflux esophagitis, hypothyroidism, rhinosinusitis, pharyngitis, anxiety, depression). Data on comorbidities were obtained through both self‐reporting and medical records. CMD is defined as one or more events of type 2 diabetes, hyperlipidemia, hypertension, coronary heart disease, and heart failure in this study.^19^, ^20^ Weight and height were also obtained through medical records. Body mass index (BMI) was calculated as weight (kg)/height (m^2^). Neck circumference was measured using a tape measure at the cricoid level.

### 2.3 Sleep assessment

A symptom questionnaire was applied to evaluate the participant's insomnia, upper respiratory symptoms, night sweats, energy during the day, and degree of daytime sleepiness. Details about the symptom assessment questionnaire are available in (Supporting Information: Appendix A, Table A.1). Daytime sleepiness was assessed with the Epworth Sleepiness Scale (ESS). These questions were selected by matching questions from our questionnaire to those of previous publications^11^, ^12^, ^13^ identifying OSA symptom subtypes. The questionnaires were answered by the participants themselves, and all questionnaires were interviewed by an investigator.

A portable monitor (Alice PDx or Alice NightOne, Philips Respironics) was used to record the in‐home polysomnography of the participants in a single overnight study. We used the American Academy of Sleep Medicine scoring manual version 2.3^21^ to diagnose sleep apnea and calculated the oxyhemoglobin desaturation index, defined as the average number of desaturation episodes per hour. Apnea was scored when there was a ≥90% decrease in airflow from the pre‐event baseline for ≥10 s; hypopnea was defined as a reduction of ≥30% in airflow from the pre‐event baseline for ≥10 s with either oxygen desaturation ≥3% or an arousal. OSA was diagnosed when >50% of respiratory events were of obstructive or mixed types. Central sleep apnea syndrome was diagnosed when ≥50% of respiratory events were of the central type. Apnea‐hypopnea index (AHI) was calculated as the mean number of apneas and hypopneas per hour of sleep. We defined OSA as ≥5 apneas and/or hypopneas per sleep hour (AHI ≥ 5 events/h). Total time with oxyhemoglobin saturation below 90% (CT90) and mean oxygen saturation (mSpO_2_) were obtained from reports.

### 2.4 Statistical analysis

Data are summarized as means and standard deviations (SD) for continuous variables, and frequencies or percentages for categorical variables. An LCA was performed among patients with OSA (AHI ≥ 5 events/h) using 10 clinically relevant variables plus the ESS ≥ 10,^12^, ^22^ reflecting questions similar to prior publications^11^, ^18^, ^23^, ^24^ on symptom clusters. The optimal model was considered according to the theory of the model and evaluated for clinical interpretation.^25^ The differences between clinical subtypes of OSA and demographic characteristics or prevalent diseases were assessed using ANOVA and *χ* ^2^ tests. Associations between the clinical subtypes of OSA and prevalent diseases were assessed using adjusted logistic regression. Statistical significance was set at *p* < .05. Mplus 8.6^18^ and SPSS 20.0 (IBM Corp) were used.

## 3 RESULTS

### 3.1 Characteristics and prevalent diseases of patients with OSA

A total of 1483 patients with OSA and their available characteristic data were included in the present study.

The characteristics are shown in Table 1. Patients with OSA had a mean (SD) age of 50.1 (13.8) years, a mean BMI of 28.0 (4.6) kg/m^2^, and most were men (75.6%). Participants had moderate OSA on average, with an AHI of 28.8 (20.8) events/h and an average ESS score of 11.5 (5.3). The mean SpO2 and CT 90% values are shown in Table 1.

**Table 1:** Characteristics and prevalent diseases of patients with OSA

Variable	OSA
Subjects *N*	1483
Men, *N* (%)	1121 (75.6)
Age, years	50.1 ± 13.8
BMI, kg/m2	28.0 ± 4.6
NC, cm	41.0 ± 3.8
ESS score	11.5 ± 5.3
AHI, events/h	28.8 ± 20.8
CT90%	9.9 ± 16.9
mSpO_2_, %	93.5 ± 2.8
Type 2 diabetes, *N* (%)	259 (17.5)
Hyperlipidemia, *N* (%)	655 (44.2)
Hypertension, *N* (%)	807 (54.4)
CHD, *N* (%)	252 (17.0)
Arrhythmia, *N* (%)	128 (8.6)
HF, *N* (%)	80 (5.4)
Stroke, *N* (%)	128 (8.6)
Asthma, *N* (%)	56 (3.8)
COPD, *N* (%)	81 (5.5)
RE, *N* (%)	182 (12.3)
Hypothyroidism, *N* (%)	30 (2.0)
Rhinosinusitis, *N* (%)	427 (28.8)
Pharyngitis, *N* (%)	494 (33.3)
Anxiety, *N* (%)	47 (3.2)
Depression, *N* (%)	15 (1.0)

*Note*: Data are presented as mean ± SD and in positive form, unless otherwise stated.

Abbreviations: AmSpO2, mean oxygen saturation; BMI, body mass index; CHD, coronary heart disease; COPD, chronic obstructive pulmonary disease; CT90, total time with oxyhemoglobin saturation below 90%; ESS, Epworth Sleepiness Scale; HF, heart failure; HI, apnea‐hypopnoea index; N, sample size; NC, neck circumference; OSA, obstructive sleep apnea; RE, reflux esophagitis; SD, standard deviation.

Table 1 shows CMD diseases prevalent in patients with OSA. Individuals with OSA had information on the prevalence of type 2 diabetes (17.5%), hyperlipidemia (44.2%), hypertension (54.4%), CHD (17.0%), heart failure (5.4%), and so on.

### 3.2 Clinical subtypes in patients with OSA

The LCA identified four clinical subtypes of OSA based on symptoms experienced by individuals diagnosed with OSA (Supporting Information: Figure A.2); Supporting Information: Table A.2 shows the details.

**Figure 1:**
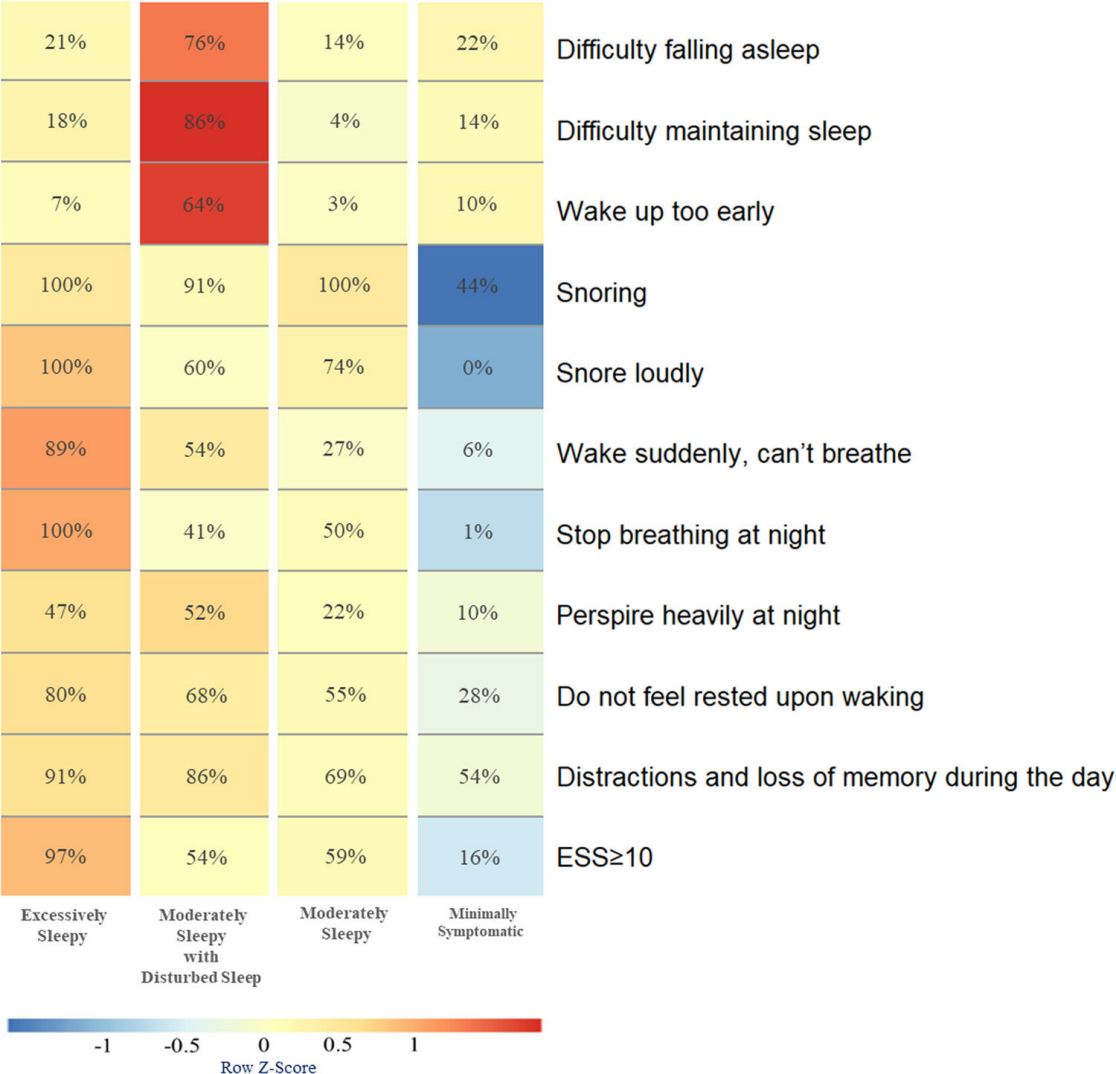
Symptom profile of the identified OSA clinical subtypes. The relative differences in symptom burden among subtypes are shown by the color scale, which represents the standardized (*z*‐score) symptom proportion or ESS ≥ 10 across groups. Brighter red indicates a higher relative symptom burden. ESS, Epworth Sleepiness Scale; OSA, obstructive sleep apnea.

Figure 1 shows the relative proportions of each symptom and ESS scores ≥10 across the clinical subtypes. Based on the distribution of observed symptoms, the subtypes were labeled as excessively sleepy (ES; *N* = 352; 24%), moderately sleepy with disturbed sleep (ModSwDS; *N* = 182; 12%), moderately sleepy (ModS; *N* = 826; 56%), and minimally symptomatic (MinS; *N* = 123; 8%).

Table 2 summarizes the characteristics of the clinical subtypes. The ES subtype had a higher BMI (28.9 ± 4.8 kg/m^2^), average ESS score (15.4 ± 3.9), AHI (38.0 ± 23.6 events/h), and CT 90% (14.9 ± 19.5) compared with the other subtypes. Further, ES had a higher proportion of men (84.9%) and neck circumference (41.6 ± 3.7 cm) than the other subtypes.

**Table 2:** Characteristics according to OSA clinical subtypes

Variable	ES (very high‐risk)	ModSwDS (high‐risk)	ModS (medium‐risk)	MinS (low‐risk)	*p* Value
Subjects, *N*	352	182	826	123	
Men, *N* (%)	299 (84.9)	95 (52.2)	646 (78.2)	81 (65.9)	<.001^a^^,^ b ^,^ c ^,^ d ^,^ e ^,^ f
Age, years	48.5 ± 12.5	59.0 ± 12.0	47.9 ± 13.6	56.1 ± 14.4	<.001^b^^,^ c ^,^ e ^,^ f
BMI, kg/m^2^	28.9 ± 4.8	27.1 ± 3.7	28.0 ± 4.6	27.6 ± 4.3	<.001^f^^,^ ^b^ ^,^ c ^,^ ^a^
NC, cm	41.6 ± 3.7	39.8 ± 3.7	41.1 ± 3.8	40.6 ± 4.0	<.001^f^^,^ b ^,^ c ^,^ a
ESS score	15.4 ± 3.9	11.0 ± 5.6	10.8 ± 4.8	5.7 ± 4.4	<.001^f^^,^ d ^,^ c ^,^ a ^,^ e
AHI, events/h	38.0 ± 23.6	23.4 ± 16.3	27.2 ± 19.7	20.5 ± 15.6	<.001^f^^,^ b ^,^ c ^,^ a ^,^ e
CT90%	14.9 ± 19.5	8.5 ± 14.9	8.0 ± 14.9	10.4 ± 21.6	<.001^f^^,^ c ^,^ a
mSpO_2_, %	92.8 ± 3.3	93.7 ± 2.3	93.9 ± 2.6	93.2 ± 3.1	<.001^f^^,^ a ^,^ e

*Note*: *p* Value from ANOVA and *χ* ^2^ test comparing variable across subtypes. The data is presented in mean ± SD unless otherwise specified. Significant differences in pairwise comparisons (*p* < .05).

Abbreviations: ES, excessively sleepy; MinS, minimally symptomatic; ModS, moderately sleepy; ModSwDS, moderately sleepy with disturbed sleepy; mSpO2, mean oxygen saturation; N, sample size; NC, neck circumference; OSA, obstructive sleep apnea; SD, standard deviation.

a ES vs. ModS.

^b^ ModSwDS vs. ModS.

^c^ ES vs. MinS.

^d^ ModSwDS vs. MinS.

^e^ ModS vs. MinS.

^f^ ES vs. ModSwDS.

In addition, ModS had a higher proportion of men (78.2%) than ModSwDS and MinS. However, ModSwDS had a higher average age (59.0 ± 12.0 years) than the ModS and ES; ModS had a lower average age (47.9 ± 13.6 years) than MinS. Furthermore, ModS had a higher neck circumference (41.1 ± 3.8 cm) and AHI (27.2 ± 19.7 events/h) than ModSwDS. MinS had a lower average ESS score (5.7 ± 4.4) than the other subtypes.

The results underscore the fact that OSA patients with clinically similar disease and demographic characteristics present distinct clinical subtypes.

### 3.3 Preliminary identification of prevalent CMD of clinical subtypes in patients with OSA

We investigated whether there were differences in the prevalence of CMD coinciding with the four clinical subtypes. We found a significant difference (Table 3) in the prevalence of type 2 diabetes (*p* < .001), hyperlipidemia (*p* < .001), hypertension (*p* = .010), CHD (*p* < .001), and HF (*p* < .001) among the four clinical subtypes, with a higher proportion of cases with hypertension (61.5%) coinciding with ES than other subtypes. Moreover, ES had a higher proportion of cases with type 2 diabetes (24.7%), hyperlipidemia (53.4%), and CHD (25.0%) than ModS (*p* < .050) and MinS (*p* < .050). ModSwDS had a higher proportion of cases with type 2 diabetes (24.7%) and CHD (21.4%) than ModS (*p* < .050) and MinS (*p* < .050), and of hyperlipidemia (45.1%) and hypertension (58.2%) than MinS (*p* < .050).

**Table 3:** Prevalence of diseases according to OSA clinical subtypes with CMD risk

Prevalent disease (%)	ES (very high‐risk) *N* = 352	ModSwDS (high‐risk) *N* = 182	ModS (medium‐risk) *N* = 826	MinS (low‐risk) *N* = 123	*p* Value
Type 2 diabetes, *N*	87 (24.7)	45 (24.7)	112 (13.6)	15 (12.2)	<.001^a^^,^ ^b^ ^,^ ^c^ ^,^ ^d^
Hyperlipidemia, *N*	188 (53.4)	82 (45.1)	348 (42.1)	37 (30.1)	<.001^a^^,^ ^b^ ^,^ ^c^ ^,^ e
Hypertension, *N*	213 (61.5)	106 (58.2)	431 (52.2)	57 (46.3)	=.010^c^ ^,^ a ^,^ ^b^
CHD, *N*	88 (25.0)	39 (21.4)	112 (13.6)	13 (10.6)	<.001^d^^,^ c ^,^ a ^,^ b
HF, *N*	13 (3.7)	22 (12.1)	35 (4.2)	10 (8.1)	<.001^f^^,^ d ^,^ b
Asthma, *N*	12 (3.4)	13 (7.1)	23 (2.8)	8 (6.5)	=.014^d^^,^ e
COPD, *N*	17 (4.8)	21 (11.5)	35 (4.2)	8 (6.5)	<.001^f^^,^ d
RE, *N*	56 (15.9)	30 (16.5)	84 (10.2)	12 (9.8)	=.010^d^^,^ a
Rhinosinusitis, *N*	134 (38.1)	36 (19.8)	234 (28.3)	23 (18.7)	<.001^f^^,^ d ^,^ a ^,^ b ^,^ e
Pharyngitis, *N*	152 (43.2)	54 (29.7)	268 (31.8)	25 (20.3)	<.001^f^^,^ a ^,^ b ^,^ e
Anxiety, *N*	10 (2.8)	17 (9.3)	16 (1.9)	4 (3.3)	<.001^d^^,^ c ^,^ f
Stroke, *N*	28 (8.0)	25 (13.7)	65 (7.9)	10 (8.1)	=.076
Arrhythmia, *N*	32 (9.1)	25 (13.7)	62 (7.5)	9 (7.3)	>.05
Hypothyroidism, *N*	12 (3.4)	3 (1.6)	14 (1.7)	1 (0.8)	>.05
Depression, *N*	4 (1.1)	1 (0.5)	7 (0.8)	3 (2.4)	>.05

*Note*: *p* Value from *χ* ^2^ test comparing variable across subtypes. The data is presented in positive form unless otherwise specified. Significant differences in pairwise comparisons (*p* < .05).

Abbreviations: CHD, coronary heart disease; CMD, cardiovascular and metabolic diseases; COPD, chronic obstructive pulmonary disease; ES, excessively sleepy; HF, heart failure; MinS, minimally symptomatic; ModS, moderately sleepy; ModSwDS, moderately sleepy with disturbed sleepy; N, sample size; OSA, obstructive sleep apnea; RE, reflux esophagitis.

^a^ ES vs. ModS.

^b^ ES vs. MinS.

^c^ ModSwDS vs. MinS.

^d^ ModSwDS vs. ModS.

^e^ ModS vs. MinS.

^f^ ES vs. ModSwDS.

The results of the logistic regression models are presented in Table 4. In adjusted models, clinical subtypes were significantly associated with type 2 diabetes, hyperlipidemia, hypertension, CHD, and HF. In between‐group comparisons (Table 4), ES was associated with a higher risk of hyperlipidemia and CHD than other subtypes (*p* < .050). ModSwDS was associated with an increased risk of HF compared to ModS (*p* = .001) and ES (*p* = .001). ES was associated with an increased risk of type 2 diabetes compared to ModS (*p* < .001) and MinS (*p* < .001). Moreover, ES was associated with a higher risk of hypertension than MinS (*p* < .001). Lastly, the ModSwDS subtype was also associated with a higher risk of type 2 diabetes (*p* = .014), hyperlipidemia (*p* = .013), and CHD (*p* = .027) than MinS.

**Table 4:** Summary of the results of the adjusted logistic regression models assessing the association between OSA clinical subtypes and CMD

Prevalent	Pairwise comparison	Prevalent (OR [95% CI])	*p* Value
Type 2 diabetes	ES vs. ModS	2.01 (1.44, 2.81)	**.000**
	ModSwDS vs. ModS	1.27 (0.83, 1.95)	.271
	ModS vs. MinS	1.8 (0.99, 3.28)	.055
	ES vs. ModSwDS	1.58 (1.00, 2.51)	.052
	ES vs. MinS	3.62 (1.94, 6.75)	**.000**
	ModSwDS vs. MinS	2.29 (1.18, 4.43)	**.014**
Hyperlipidemia	ES vs. ModS	1.47 (1.13, 1.90)	**.004**
	ModSwDS vs. ModS	0.89 (0.63, 1.26)	.517
	ModS vs. MinS	2.11 (1.38, 3.22)	**.001**
	ES vs. ModSwDS	1.65 (1.12, 2.42)	**.011**
	ES vs. MinS	3.09 (1.96, 4.87)	**.000**
	ModSwDS vs. MinS	1.88 (1.14, 3.08)	**.013**
Hypertension	ES vs. ModS	1.29 (0.98, 1.70)	.071
	ModSwDS vs. ModS	0.79 (0.55, 1.13)	.197
	ModS vs. MinS	1.97 (1.30, 2.97)	**.001**
	ES vs. ModSwDS	1.63 (1.09, 2.45)	**.018**
	ES vs. MinS	2.53 (1.61, 3.97)	**.000**
	ModSwDS vs. MinS	1.55 (0.95, 2.53)	.080
CHD	ES vs. ModS	2.13 (1.51, 3.00)	**.000**
	ModSwDS vs. ModS	0.94 (0.60, 1.47)	.793
	ModS vs. MinS	2.34 (1.24, 4.42)	**.009**
	ES vs. ModSwDS	2.26 (1.39, 3.66)	**.001**
	ES vs. MinS	4.98 (2.57, 9.63)	**.000**
	ModSwDS vs. MinS	2.20 (1.09, 4.45)	**.027**
HF	ES vs. ModS	0.72 (0.37, 1.40)	.335
	ModSwDS vs. ModS	2.77 (1.50, 5.12)	**.001**
	ModS vs. MinS	0.57 (0.27, 1.22)	.148
	ModSwDS vs. ES	3.84 (1.78, 8.28)	**.001**
	ES vs. MinS	0.41 (0.17, 1.00)	.050
	ModSwDS vs. MinS	1.58 (0.70, 3.56)	.268

*Note*: According to the outcome, logistic regression model adjusted for age, sex, BMI. Bold p‐value means that there are statistical significances *p* < .05.

Abbreviations: CI, confidence interval; CHD, coronary heart disease; CMD, cardiovascular and metabolic diseases; ES, excessively sleepy; HF, heart failure; MinS, minimally symptomatic; ModS, moderately sleepy; ModSwDS, moderately sleepy with disturbed sleepy; OR, odds ratio; OSA, obstructive sleep apnea.

## 4 DISCUSSION

This study applied LCA to analyze the potential subtypes of OSA in Chinese patients based on symptoms and identified four types of clinical subtypes: ES (*N* = 352; 24%), ModS (*N* = 826; 56%), ModSwDS (*N* = 182; 12%), and MinS (*N* = 123; 8%). The ES subtype had a higher proportion of men, BMI, neck circumference, average ESS score, AHI, and CT 90% compared with the other subtypes. MinS had a lower average ESS score than the other subtypes. Further, ES, ModSwDS, ModS, and MinS subtypes are very high‐risk, high‐risk, medium‐risk, and low‐risk in prevalent CMD.

In the study, patients were analyzed in order of potential categories according to the 2–10 class model. A single subgroup began to appear in the 5‐class method (Supporting Information: Figure A.3), which only included 46 subjects (3%); therefore, 5‐class and above models were not considered. In the 2‐class and 3‐class methods, the subgroups still included patients with significantly different clinical characteristics. For example, in the 3‐class method, patients with moderate sleepiness exhibited both severe upper respiratory symptoms (snoring, loudness of snoring, and respiratory arrest at night) and mild upper respiratory symptoms. Thus, the clinical manifestations were completely different and needed to be further subdivided. A statistical analysis of the prevalent CMD according to each subtype of the 4‐class method found that there were significant differences in the prevalence of CMD between the clinical subtypes of the 4‐class methods. The 5‐class method had more subtype groups, the application was more cumbersome. Based on the comprehensive statistical results and clinical significance, the 4‐class model is the best clinical subtype. See Figure 1, members of the subtype defined as “Moderately Sleepy with Disturbed Sleep” had the highest probability of experiencing insomnia‐related symptoms, including difficulty falling asleep (76%), waking up too early (64%), and most prominently, difficulty maintaining sleep (86%). Perspiring heavily at night (52%) was also prominent. For the majority of symptoms, the probability was markedly lower in the subtype that was “Minimally Symptomatic” than in other subtypes. A subtype was the “Excessively Sleepy,” with a markedly higher probability of ESS ≥ 10 (97%) and complaining of sleepiness‐related symptoms, such as snoring (100%), snoring loudly (100%), Waking suddenly, can't breathe (89%), Stopping breathing at night (100%), do not feel rested upon waking (80%), and distractions and loss of memory during the day (91%). The last subtype was defined as “Moderately Sleepy” according to the moderate burden of symptoms.

There are significant differences in the prevalence of CMD among the clinical subtypes. ES has the highest prevalence of type 2 diabetes, hyperlipidemia, hypertension, and CHD, while MinS has the lowest prevalence of CMD. Further, ModSwDS has a higher prevalence of type 2 diabetes, hyperlipidemia, hypertension, and CHD than ModS. The results indicate that there were significant differences in the prevalence of CMD among Chinese patients diagnosed with one of the four clinical subtypes of OSA. In terms of the risk of combined CMD, ES is a very high‐risk subtype, ModSwDS is a high‐risk subtype, ModS is a medium‐risk subtype, and MinS is a low‐risk subtype.

The present study found that the prevalence of CMD in ModSwDS was higher than that in ModS. This may be because insomnia can increase the excitability of the sympathetic nerve and the sympathetic‐adrenal medulla system hyperactivity, causing the level of catecholamines to rise, and increasing the burden on the circulatory system^3^, ^26^, ^27^; insomnia can also cause excessive activation of the hypothalamus‐pituitary‐adrenergic axis, causing abnormal endocrine metabolism.^26^, ^28^, ^29^ There was no significant difference in ESS between the two clinical subtypes. Considering the difference in the prevalence of CMD between the two subtypes, clinical attention should be paid to the symptoms of insomnia in patients with OSA, and suitable treatment should be provided. Previous studies^9^, ^10^ have found that CPAP treatment does not significantly improve the cardiovascular prognosis of patients with OSA. This is because CPAP treatment cannot effectively control the cardiovascular and metabolic system damage caused by insomnia.^30^

This study found that it is one‐sided to assess the severity of OSA in patients based on upper respiratory symptoms alone. Mild upper respiratory symptoms do not mean that patients with OSA have a lower prevalence of CMD. The symptoms of snoring and stopped breathing at night in ModSwDS are lower than those in ModS, and the symptom of loud snoring is also lower in ModSwDS than in ModS, but the prevalence of CMD in ModSwDS is higher than that in ModS. Our research results show that even if the upper respiratory symptoms of OSA patients are mild, patients with CMD should be fully evaluated to avoid missed diagnoses and misjudgments about patients' conditions.

There were significant differences in the prevalence of type 2 diabetes, hyperlipidemia, hypertension, CHD, and HF among the subtypes in our study (*p* < .05), and there was no significant difference in the prevalence of stroke (*p* > .05). These results are different from the results of the SHHS^12^ study concerning HF, CHD, and stroke. In addition, the proportion of MinS in our study was 8%, which was lower than that in the SHHS^12^ study (33%). This may have been caused by the different samples under investigation. Further, the SHHS^12^ study is community‐based. Our research focused on patients undergoing sleep monitoring at sleep centers. The two studies also exhibit differences in the age and race of the participants. The participants of the SHHS study (AHI ≥ 15) were older adults (66.0 ± 10.5 years), primarily European (86.1%) and African (9.0%) Americans. The participants of our study were Chinese middle‐aged patients (50.1 ± 13.8 years), experiencing mild, moderate, or severe OSA, compensating for the limitations of the previous study and expanding the study object to patients with all severity levels.

The present study has several strengths, such as a cluster of OSA participants based on clinical symptoms in Chinese from a large sample, and the use of adults of all OSA severity levels, allowing be applied to all patients with OSA. Future investigations in Cardiovascular risk among different OSA subtypes would be more powered. Our study had several limitations. First, this was a single‐center study, and only included patients diagnosed with OSA from the Sleep Center of Beijing Anzhen Hospital, potentially causing a referral bias. Second, the research participants were all diagnosed with OSA through in‐home polysomnography, which may underestimate the severity of the disease. Third, our study is a cross‐sectional study that did not observe long‐term results.

In conclusion, our study identified four clinical subtypes of OSA in Chinese patients and found that the prevalence of CMD coinciding with each clinical subtype is different. This enables the administration of more accurate treatments for patients with different clinical manifestations. Further research will be conducted to observe whether different clinical subtypes predict different clinical outcomes and whether they can predict the incidence of CMD in the future.

## CONFLICT OF INTEREST

The authors declare no conflict of interest.

## Supporting information

Supplementary information.

## Data Availability

Data sharing is not applicable.
