# Supplementary material for: Differences in the prevalence of cardiovascular and metabolic diseases coinciding with clinical subtypes of obstructive sleep apnea
Source: Clin Cardiol. 2022 Nov 20;46(1):92–9. doi: 10.1002/clc.23941 (PMC9849430; doi:10.1002/clc.23941)
Supplement: Supplementary file 1 — Supplementary information. [file CLC-46-92-s001.pdf]

Differences in the Prevalence of Cardiovascular and Metabolic Diseases Coinciding with Clinical Subtypes of Obstructive Sleep Apnea

Yang Gao[a], Yaxin Guo[d], Jiajia Dong[a], Yifan Liu[a,c], Wen Hu[a], Mi Lu[a], Yueran Shen[d], Yi Liu[a], Yongxiang Wei[a,c], Zhenlin Wang[b,*], Xiaojun Zhan[a,c,*]

a Department of Otolaryngology-Head and Neck Surgery, Beijing Anzhen Hospital, Capital Medical University, Beijing, China

b Department of Otolaryngology-Head and Neck Surgery, Xuanwu Hospital Capital Medical University, Beijing, China

c Department of Otorhinolaryngology-Head and Neck Surgery, Capital Institute of Pediatrics, Beijing, China

d Beijing Institute of Heart Lung and Blood Vessel Diseases, Beijing, China

Online Data Supplement

Statistical analysis

Class analysis was performed among patients with OSA (AHI≥5 events/h), using a latent class analysis (LCA) with questionnaire data on relevant symptoms, similar to previous publications [1-4]. We used Mplus8.6 to estimate the model. The optimal number of classes was determined by running LCA to obtain solutions from 2 to 10 classes, and comparing the resulting Bayesian Information Criterion (BIC) values (Figure A.2) and LMR p-value (Table A.2). The solution with the lowest BIC value was considered the optimal number of classes [5]. The LMR p-value less than 0.05 means that the current classification quantity is different from the model with one less classification quantity in the different tests [6]. Entropy<0.60 indicates that >20% of cases are classified falsely and Entropy>0.80 indicates that >90% of cases are classified correctly [7]. Researchers believe that the choice of multiple models relies heavily on sampling results, and models with suboptimal data results are often selected in research [8]. Although the solution with 5 classes showed the lowest BIC (Figure A.1) and is significantly different from the solution with 4 classes (Table A.1), we ultimately chose the solution with 4 classes as the optimal model.

Table A.1.Description of the symptom questions for participant.

	Question	Response	
1	Difficulty falling asleep?	No☐	Yes☐
2	Difficulty maintaining asleep?	No☐	Yes☐
3	Do you wake up early in the morning and are unable to fall back asleep?	No☐	Yes☐
4	Do you snore?	No☐	Yes☐
	Is the snoring loud (louder than talking)?	No☐	Yes☐
5	Do you wake suddenly and feel as if you cannot breathe during sleep?	No☐	Yes☐
6	Do you perspire heavily at night?	No☐	Yes☐
7	Do you stop breathing at night?	No☐	Yes☐
8	Do you not feel rested upon waking?	No☐	Yes☐
9	Distractions and loss of memory during the day?	No☐	Yes☐
10	Epworth Sleepiness Scale score ≥10	No☐	Yes☐

Table A.2. Latent Class Model Fit Comparison (N=1483).

Number of Classes	Df	BIC	Entropy	LMR p-value	FftSC
2	23	17524	0.64	< 0.01	29%
3	35	17270	0.70	< 0.01	12%
4	47	17173	0.73	< 0.01	8%
5	59	17159	0.73	< 0.01	3%
6	71	17178	0.70	=0.03	3%
7	83	17218	0.70	=0.16	3%
8	95	17256	0.71	=0.24	3%
9	107	17306	0.71	=0.55	2%
10	119	17383	0.72	=0.57	2%

Abbreviations: Df: degrees of freedom; BIC: Bayesian Information Criterion; LMR: Lo-Mendell-Rubin; FftSC: frequency for the smallest class

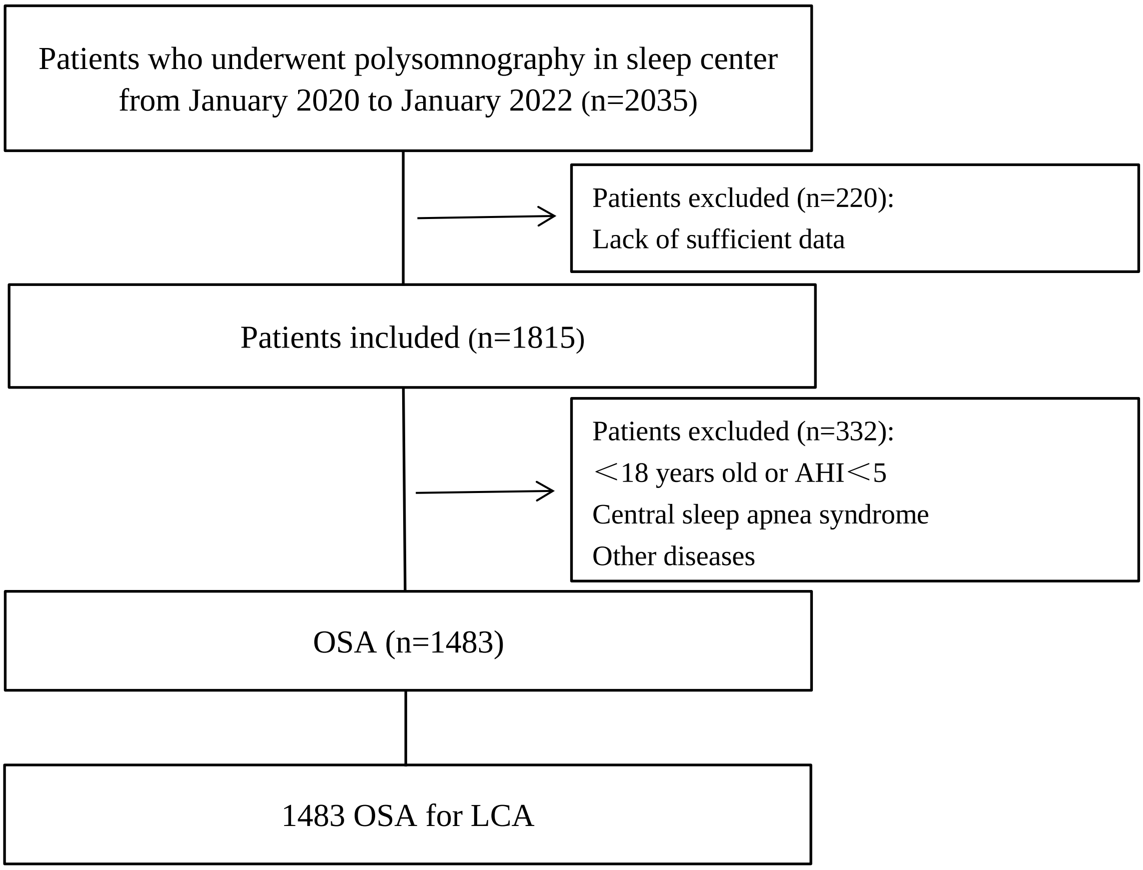

Other diseases:

1. Severely impaired consciousness
2. Previous history of malignancy

Figure A.1. A flow diagram showing the analysis of patients with OSA who underwent polysomnography to determine clinical subtypes. The patients were classified as OSA if apnea-hypopnea index (AHI) ≥ 5 events/h.

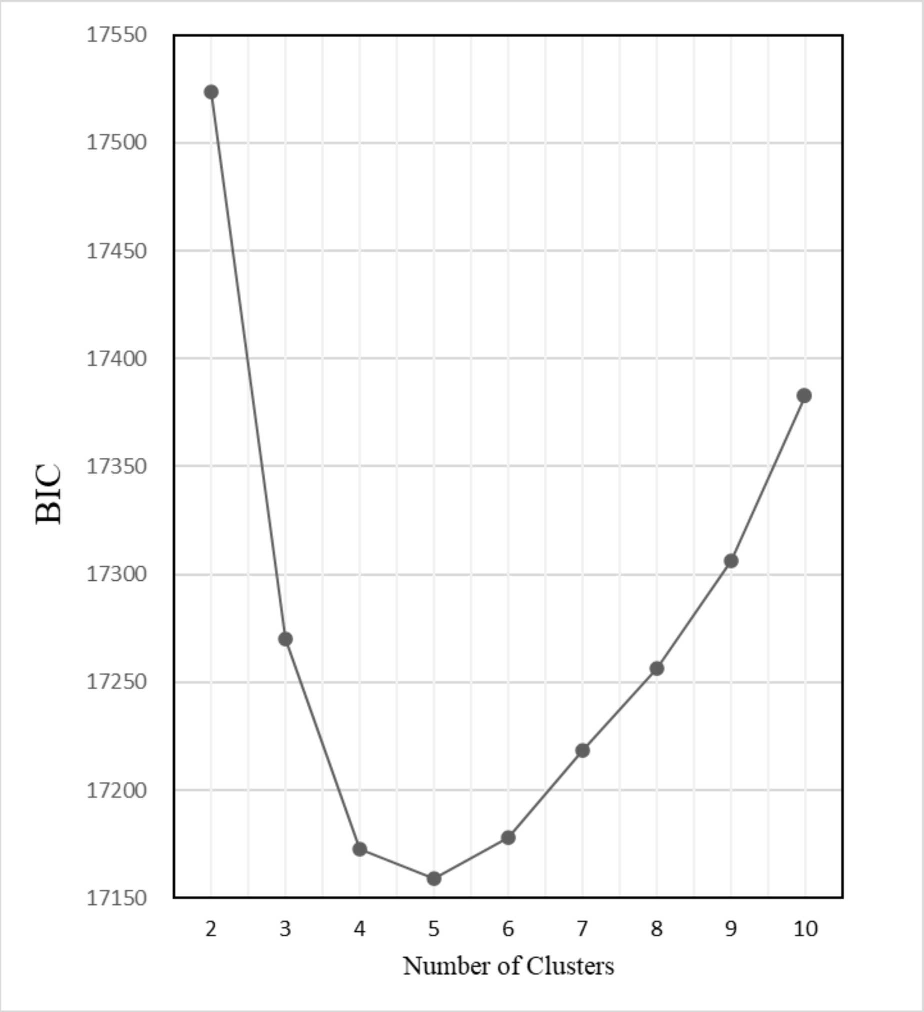

Figure A.2. Comparison of the Bayesian Information Criteria statistics for Latent Class Analysis results.

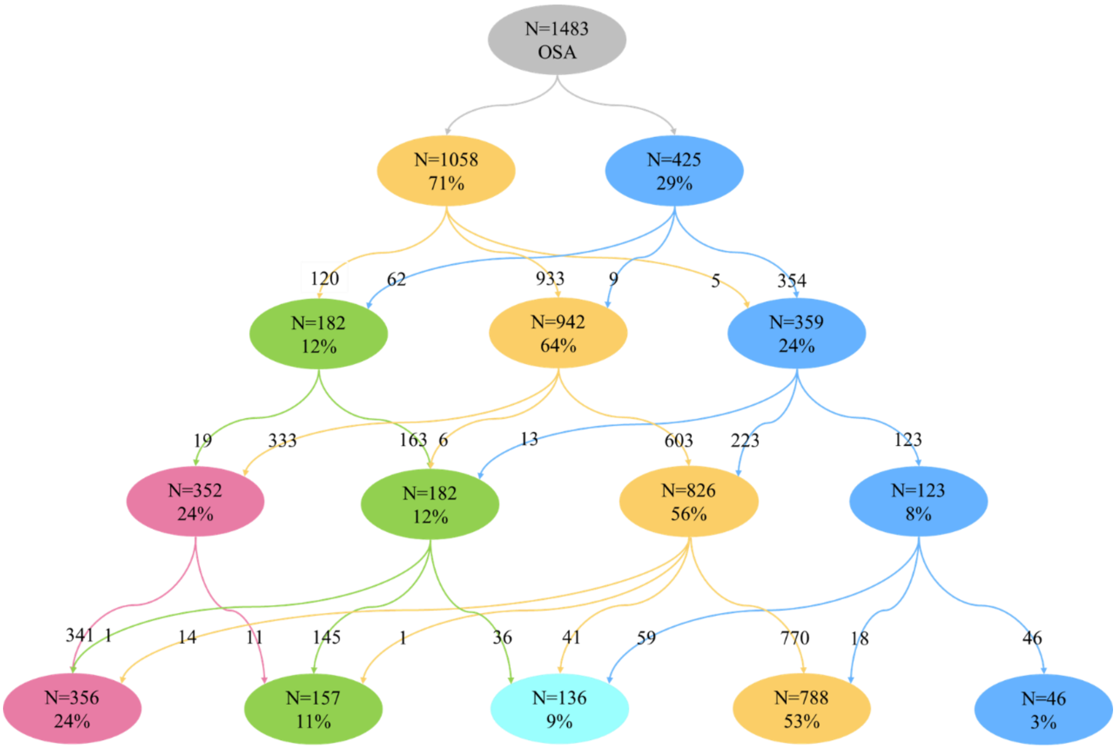

Figure A.3. Assignment of 1483 patients into distinct classes over a sequence of the latent class model with two, three, four, and five classes. Ellipses show class membership whilst the values along the arrow represent the number of patients moving from one class to another in models with increasing classes.

References:
[1] Ye L, Pien GW, Ratcliffe SJ, Björnsdottir E, Arnardottir ES, Pack AI, et al. The different clinical faces of obstructive sleep apnoea: a cluster analysis. EUR RESPIR J. 2014;44: 1600-7.
[2] Keenan BT, Kim J, Singh B, Bittencourt L, Chen N, Cistulli PA, et al. Recognizable clinical subtypes of obstructive sleep apnea across international sleep centers: a cluster analysis. Sleep (New York, N.Y.). 2018;41: 1.
[3] Kim J, Keenan BT, Lim DC, Lee SK, Pack AI, Shin C. Symptom-Based Subgroups of Koreans With Obstructive Sleep Apnea. J CLIN SLEEP MED. 2018;14: 437-43.
[4] Pien GW, Ye L, Keenan BT, Maislin G, Björnsdóttir E, Arnardottir ES, et al. Changing Faces of Obstructive Sleep Apnea: Treatment Effects by Cluster Designation in the Icelandic Sleep Apnea Cohort. SLEEP. 2018;41: .
[5] Nylund KL, Asparouhov T, Muthén BO. Deciding on the Number of Classes in Latent Class Analysis and Growth Mixture Modeling: A Monte Carlo Simulation Study. Structural equation modeling. 2007;14: 535-69.
[6] Asparouhov T, Muthén B. Using Mplus TECH11 and TECH14 to test the number of latent classes. Mplus Web Notes: No. 14. 2012.
[7] Lubke G, Muthén BO. Performance of Factor Mixture Models as a Function of Model Size, Covariate Effects, and Class-Specific Parameters. STRUCT EQU MODELING. 2007;14(1): 26-47.
[8] Preacher KJ, Merkle EC. The problem of model selection uncertainty in structural equation modeling. PSYCHOL METHODS. 2012;17(1): 1.
